# Pregnane X Receptor Knockout Mice Display Aging-Dependent Wearing of Articular Cartilage

**DOI:** 10.1371/journal.pone.0119177

**Published:** 2015-03-06

**Authors:** Kotaro Azuma, Stephanie C. Casey, Tomohiko Urano, Kuniko Horie-Inoue, Yasuyoshi Ouchi, Bruce Blumberg, Satoshi Inoue

**Affiliations:** 1 Department of Geriatric Medicine, Graduate School of Medicine, The University of Tokyo, 7-3-1 Hongo, Bunkyo-ku, Tokyo, 113-8655, Japan; 2 Department of Developmental and Cell Biology, University of California Irvine, Irvine, CA, 92697-2300, United States of America; 3 Department of Anti-aging Medicine, Graduate School of Medicine, The University of Tokyo, 7-3-1 Hongo, Bunkyo-ku, Tokyo, 113-8655, Japan; 4 Division of Gene Regulation and Signal Transduction, Research Center for Genomic Medicine, Saitama Medical University, 1397-1 Yamane, Hidaka, Saitama, 350-1241, Japan; 5 Toranomon Hospital, 2-2-2 Toranomon, Minato-ku, Tokyo, 105-8470, Japan; Nihon University School of Medicine, JAPAN

## Abstract

Steroid and xenobiotic receptor (SXR) and its murine ortholog, pregnane X receptor (PXR), are nuclear receptors that are expressed at high levels in the liver and the intestine where they function as xenobiotic sensors that induce expression of genes involved in detoxification and drug excretion. Recent evidence showed that SXR and PXR are also expressed in bone tissue where they mediate bone metabolism. Here we report that systemic deletion of PXR results in aging-dependent wearing of articular cartilage of knee joints. Histomorphometrical analysis showed remarkable reduction of width and an enlarged gap between femoral and tibial articular cartilage in PXR knockout mice. We hypothesized that genes induced by SXR in chondrocytes have a protective effect on articular cartilage and identified Fam20a (family with sequence similarity 20a) as an SXR-dependent gene induced by the known SXR ligands, rifampicin and vitamin K2. Lastly, we demonstrated the biological significance of Fam20a expression in chondrocytes by evaluating osteoarthritis-related gene expression of primary articular chondrocytes. Consistent with epidemiological findings, our results indicate that SXR/PXR protects against aging-dependent wearing of articular cartilage and that ligands for SXR/PXR have potential role in preventing osteoarthritis caused by aging.

## Introduction

Steroid and xenobiotic receptor (SXR) and its murine ortholog pregnane X receptor (PXR) (also known as PAR and NR1I2) are nuclear receptors that are mainly expressed in the liver and intestine where they regulate transcription of drug metabolizing enzymes and transporters [1,2]. These receptors have been shown to be activated by various endogenous and dietary substances, pharmaceutical agents, and xenobiotic compounds [3].

In addition to its function as a xenobiotic sensor, we found that SXR/PXR plays important roles in bone tissue [4]. Expression of SXR/PXR was detected in osteoblasts [5], and systemic ablation of PXR caused osteopenia and consequent mechanical fragility [6], indicating a bone protective function for SXR/PXR. We previously reported that the fat soluble vitamin K2 activated SXR/PXR and elicited SXR/PXR-dependent biological functions in bone [5,7]. In clinical studies, administration of vitamin K2 was shown to prevent bone fracture [8,9], which led to the approval of vitamin K2 as a drug for osteoporosis in eastern Asian countries.

Some epidemiological studies have implied that vitamin K is related to another skeletal disease, osteoarthritis. In both North America and Japan, low vitamin K intake was shown to be related to the prevalence of osteoarthritis [10–12]. Based on these studies, we hypothesized that SXR/PXR-mediated vitamin K signaling exerts protective effects on articular cartilage and bone tissue and decided to evaluate the articular cartilage in PXR knockout mice.

In this study, we characterized the histomorphometrical phenotypes of PXR knockout mice to understand the roles of SXR/PXR in the cartilage tissue. Our results demonstrated that loss of SXR/PXR caused wearing of articular cartilage of knee joints in aged mice. We also found a candidate gene mediating the cartilage-protective effect of SXR/PXR by analysis of SXR-dependent ligand-induced genes in a murine chondrocytic cell line.

## Materials and Methods

### Ethics Statement

This study was carried out in strict accordance with the consent of the Animal Care and Use Committees of University of California, Irvine. The protocol was approved by the Animal Care and Use Committees of University of California, Irvine (Protocol Number: 2003–2487) and the Animal Experimentation Committee of the University of Tokyo (Protocol Number: P09-001).

### Animal experiments

The generation of PXR knockout (PXRKO) mice has previously been described [13]. The PXRKO mice were maintained in the 129/Sv background. The animals were housed in a temperature-controlled room (22°C) with a daily light/dark schedule of 12 h. The animals had free access to water and were fed a standard laboratory chow. Age-matched 129/Sv wild-type mice were used as controls and maintained under the same conditions. When mice were sacrificed, anesthesia with isoflurane inhalation or an intraperitoneal injection of 2.5% avertin was employed to minimize suffering of animals. Cervical dislocation was done following anesthesia to ensure death.

### Cartilage histomorphometry

The legs were fixed with 70% ethanol and soft tissues were removed. Cartilage histomorphometry was performed on undecalcified sections with the Villanueva Bone Stain [14].

### Cell culture and infection with adenovirus vectors

Primary culture of articular chondrocytes was performed as previously described [15]. Cos7 cells were purchased from ATCC (Manassas, VA, USA) and grown in Dulbecco's modified Eagle medium (DMEM) with 10% fetal calf serum (FCS) at 37°C under 5% CO2. ATDC5 cells were purchased from RIKEN Cell Bank (Tsukuba, Japan). ATDC5 cells were maintained in medium consisting of a 1:1 mixture of DMEM and Ham's F-12 (Invitrogen) supplemented with 10% FCS, at 37°C under 5% CO2. When ATDC5 cells were treated with SXR ligands, they were cultured in phenol red-free DMEM with 5% charcoal/dextran-treated FCS from 24 h prior to rifampicin (Nacalai Tesque, Kyoto, Japan), MK-4 (gifted by Eisai Co., Ltd., Tokyo, Japan), or vehicle (0.1% ethanol) treatment. Recombinant adenoviruses were generated using the Adenovirus Expression Vector Kit (Takara Bio, Otsu, Japan) and used at a multiplicity of infection (MOI) of 40–100 according to the manufacturer's protocol.

### Microarray and cluster analysis

Total RNA was extracted from the cells using the ToTALLY RNA Kit (Ambion, Austin, TX). Profiling of mRNA was performed on Affymetrix Mouse Gene 1.0 ST arrays (Affymetrix Inc., Santa Clara, USA) according to the Gene Chip labeling assay manual version 4. Hierarchical clustering of gene expression was performed using Cluster 3.0 software [16], and the heat map was created using Java Treeview version 1.1.6 [17].

### Quantitative real-time polymerase chain reaction analysis

Total RNA was isolated using the TOTALLY RNA kit (Ambion). First-strand cDNA was generated by using PrimeScript RT reagent Kit (Takara Bio, Otsu, Japan). mRNA levels were quantified by real-time polymerase chain reaction (PCR) using SYBR Green PCR master mix (Applied Biosystems, Foster City, CA, USA) and a 7500 Fast Real-Time PCR system (Applied Biosystems). Relative differences in the amount of the PCR products between the treatment groups were evaluated using GAPDH as an internal control. The primer sequences for PCR amplification were as follows: PXR Forward, 5′-TCATGTCCGATGCCGCTGTG-3′; PXR Reverse, 5′-AGGCGGTGGAGCCTCAATCT-3′; Fam20a Forward, 5′-CCGCAGACTCCAGATCATCC-3′; Fam20a Reverse, 5′-GTCTGGAGCCGAACGTTGTG-3′; GAPDH Forward, 5′-TGGCATGGCCTTCCGTGTTC-3′; GAPDH Reverse, 5′-CAGATGCCTGCTTCACCACC-3′, Col2a1 Forward, 5′-GCTGGCAGCTGTCTGCAGAA-3′; Col2a1 Reverse, 5′-CGCAGAGGACATTCCCAGTG-3′; aggrecan Forward, 5′-GGTCTTTGTGACTCTGAGGG-3′; aggrecan Reverse, 5′-AGTAGCAGGGGATGGTGAGG-3′; ADAMTS5 Forward, 5′-AAACACGGGAGCGAGGCCAT-3′; ADAMTS5 Reverse, 5′-AGGTAGCCCACTTTGCCACC-3′, Mmp-13 Forward, 5′-TGGACTCCCTGTTGGTCCCT-3′, Mmp-13 Reverse, 5′-TCCCGCAAGAGTCGCAGGAT-3′.

### Luciferase assay

Three copies of putative PXR responsive element or mutated PXR responsive element were inserted into tk-luc vector [18] by annealing complimentary oligonucleotides containing cohesive BamHI and HindII sites at both ends. Oligonucleotides used are as follows: 5’-AGCTTTGACCTTGCCCTGACCCCCATCCCGGGAAATGACCTTGCCCTGACCCCCATCCCGGGAAATGACCTTGCCCTGACCCG-3’ and 5’-GATCCGGGTCAGGGCAAGGTCATTTCCCGGGATGGGGGTCAGGGCAAGGTCATTTCCCGGGATGGGGGTCAGGGCAAGGTCAA-3’ for PXR responsive element, 5’-AGCTTTGCTGTTGCCCTGCTGCCCATCCCGGGAAATGCTGTTGCCCTGCTGCCCATCCCGGGAAATGCTGTTGCCCTGCTGCG-3’ and 5’-GATCCGCAGCAGGGCAACAGCATTTCCCGGGATGGGCAGCAGGGCAACAGCATTTCCCGGGATGGGCAGCAGGGCAACAGCAA-3’ for mutated PXR responsive element. Cos7 cells were transfected with pCDG SXR or PXR plasmid [1], tk-luc PXR responsive element or mutated PXR responsive element, and CMX-β-galactosidase control plasmid using Opti-MEM (Life technologies, Carlsbad, CA, USA) and Lipofectamine 2000 (Life technologies). The cells were stimulated with indicated ligands for 24 h, and assayed for luciferase and β-galactosidase activity. Luciferase activity was measured as relative light units generated by catalyzing luciferin, then normalized by optical density of ortho-nitrophenol generated from ortho-nitrophenyl-β-D-galactopyranoside (ONPG) catalyzed by β-galactosidase.

### Small interfering RNA

Small interfering RNA for Fam20a (sense strand, GCCAAGUGUCCGUACAUGUGC; antisense strand, ACAUGUACGGACACUUGGCGA) and control siRNA (NC; negative control) were purchased from RNAi Inc. (Tokyo, Japan). SiRNA (4 pmol) was transfected into primary articular chondrocytes (5 × 10^4^ cells/well on 24-well plates) isolated from C57BL6/J mice using 0.5 μl of siPORT NeoFX transfection agent (Ambion) using the reverse transfection method according to the manufacturer’s instructions.

### Statistical analysis

Data are expressed as mean ± SEM. Differences between mean values were analyzed using unpaired Student’s t-test unless otherwise noted. One-way analysis of variance was used with Dunnett’s test for multiple comparisons.

## Results

### Age-dependent wearing of articular cartilage in PXR knockout mice

**Fig 1:**
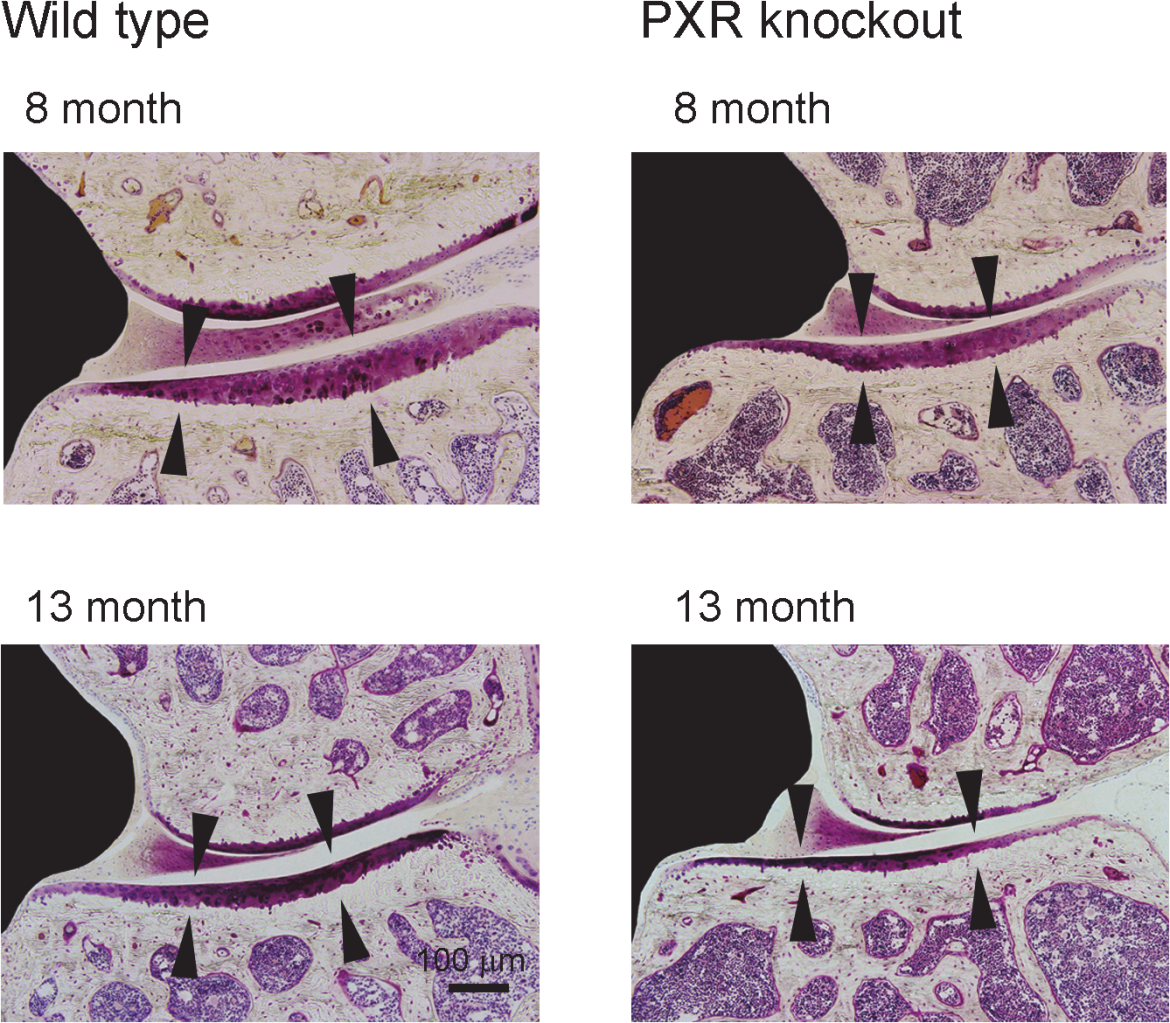
Wearing of articular cartilage of the knee joint in PXR knockout mouse. Representative microscopic image of articular cartilage of 8-month-old and 13-month-old wild-type and PXR knockout mice are shown. Arrowheads indicate lateral articular cartilage of the tibia.

**Fig 2:**
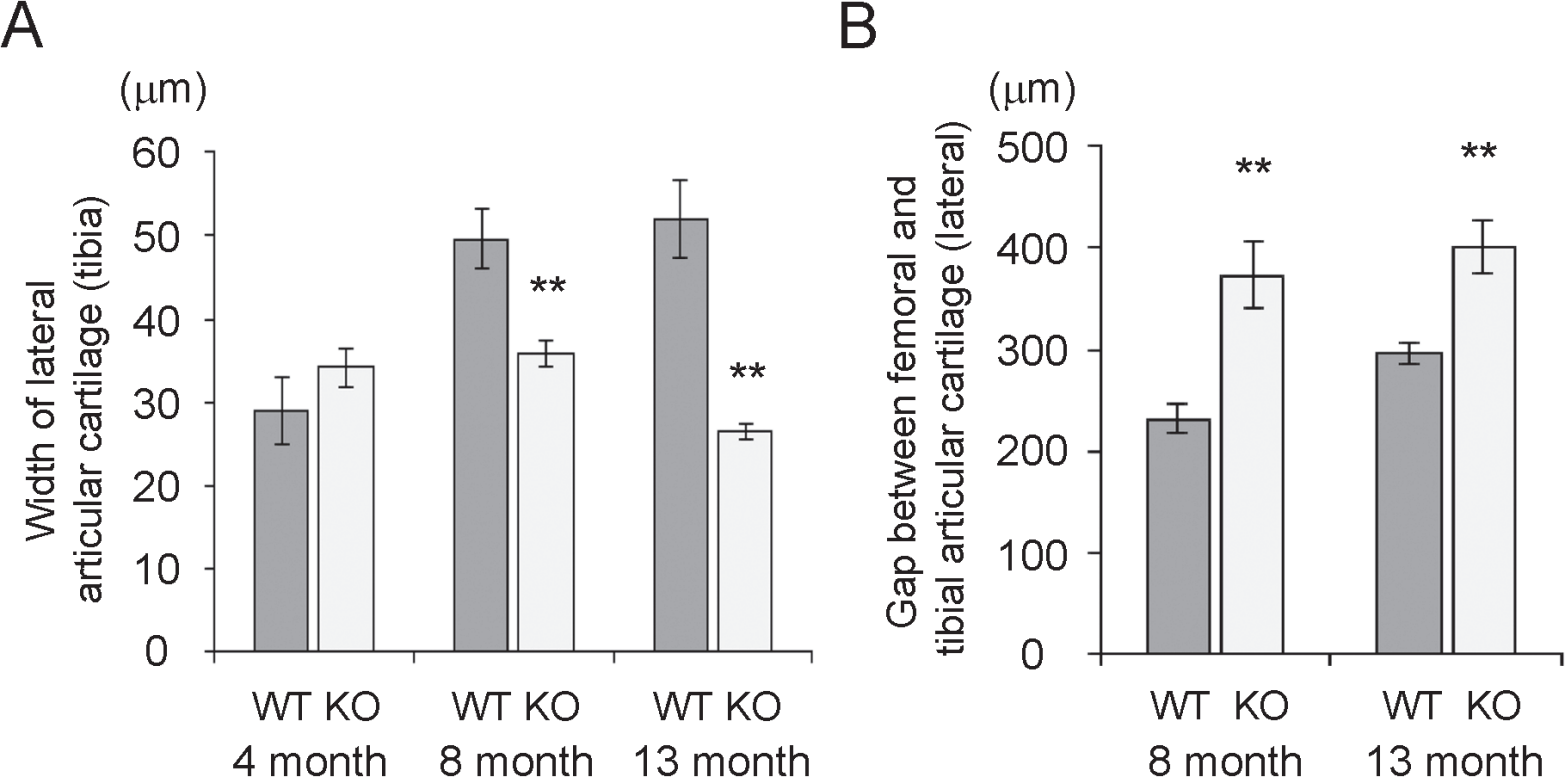
Age-dependent wearing of articular cartilage of the knee joint. (A) Width of lateral articular cartilage of the tibia in 4-month-old wild type (WT; n = 8) and PXR knockout (KO; n = 8) mice, 8 month-old wild type (WT; n = 6) and PXR knockout (KO; n = 6) mice, and 13 month-old wild type (WT; n = 5) and PXR knockout (KO; n = 4) mice is shown. (B) Gap between femoral and tibial articular cartilage of 8-month-old wild-type (WT; n = 6) and PXR knockout (KO; n = 6) mice and 13 month-old wild-type (WT; n = 5) and PXR knockout (KO; n = 4) mice are shown. **P < 0.01.

To examine the effects of PXR on the articular cartilage, we utilized PXR knockout (PXRKO) mice. We evaluated the knee joints of relatively old 8-month-old and 13-month-old female PXRKO mice and female wild-type (WT) mice of the same age (Fig. 1). Histomorphometrical analysis of the lateral articular cartilage of the tibia revealed that the cartilage width of 8-month-old and 13-month-old PXRKO mice was significantly decreased compared with that of WT mice (Fig. 2A). The gap between the femoral and tibial lateral articular cartilage was significantly increased in 8-month-old and 13-month-old PXRKO mice relative to that of WT mice (Fig. 2B). These phenotypes are similar to those of osteoarthritis seen in elderly people. We then examined when this osteoarthritis-like phenotype develops in PXRKO mice. We evaluated the width of the lateral articular cartilage of the tibia in 4-month-old female PXRKO mice and WT mice. In contrast to aged mice, 4-month-old PXRKO mice displayed no significant differences in the width of the lateral articular cartilage compared with control mice (Fig. 2A). Compared with WT mice, the thickness of the articular cartilage of PXRKO mice did not increase between 4 months and 8 months, and it began to show wear between 8 months and 13 months. These results indicate that wearing of cartilage in PXRKO mice is an age-dependent pathological process similar to human osteoarthritis, although the damage in the surface of the cartilages was milder than expected considering their decreased width.

### SXR-dependent induction of Fam20a by SXR ligands

**Fig 3:**
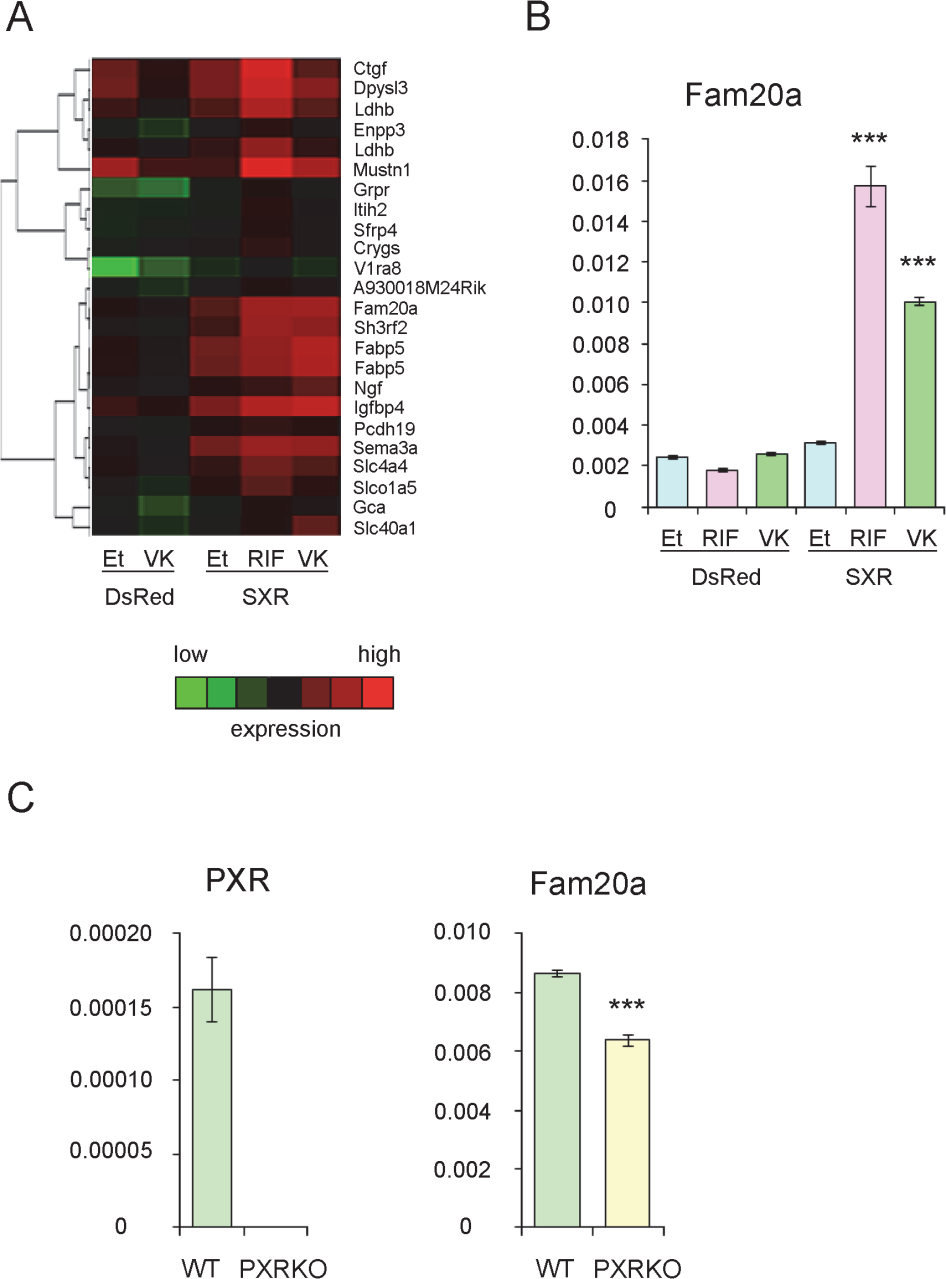
SXR-dependent induction of Fam20a by SXR ligands. (A) ATDC5 cells were infected with adeno-SXR or adeno-DsRed and cultured in phenol red-free DMEM with charcoal/dextran-treated FCS (5%) containing rifampicin (RIF) (10 μM), vitamin K2 (VK) (10 μM), or ethanol (Et). Total RNA was extracted and gene expression was analyzed by microarray followed by hierarchical cluster analysis. A heat-map visualization of clusters including SXR-dependent ligand-induced genes is shown. Red grids indicate high expression and green grids indicate low expression. (B) SXR-dependent induction of Fam20a was validated by quantitative real-time PCR. Expression of GAPDH was used as an internal control. ***P < 0.001 in Dunnett’s test with adeno-SXR-infected ethanol-treated cells as a control. (C) Expression of Fam20a was decreased in primary articular chondrocytes derived from PXR knockout mice. Primary chondrocytes were purified from the femoral and knee joints of newborn PXR knockout mice and wild type mice. ***P < 0.001.

**Fig 4:**
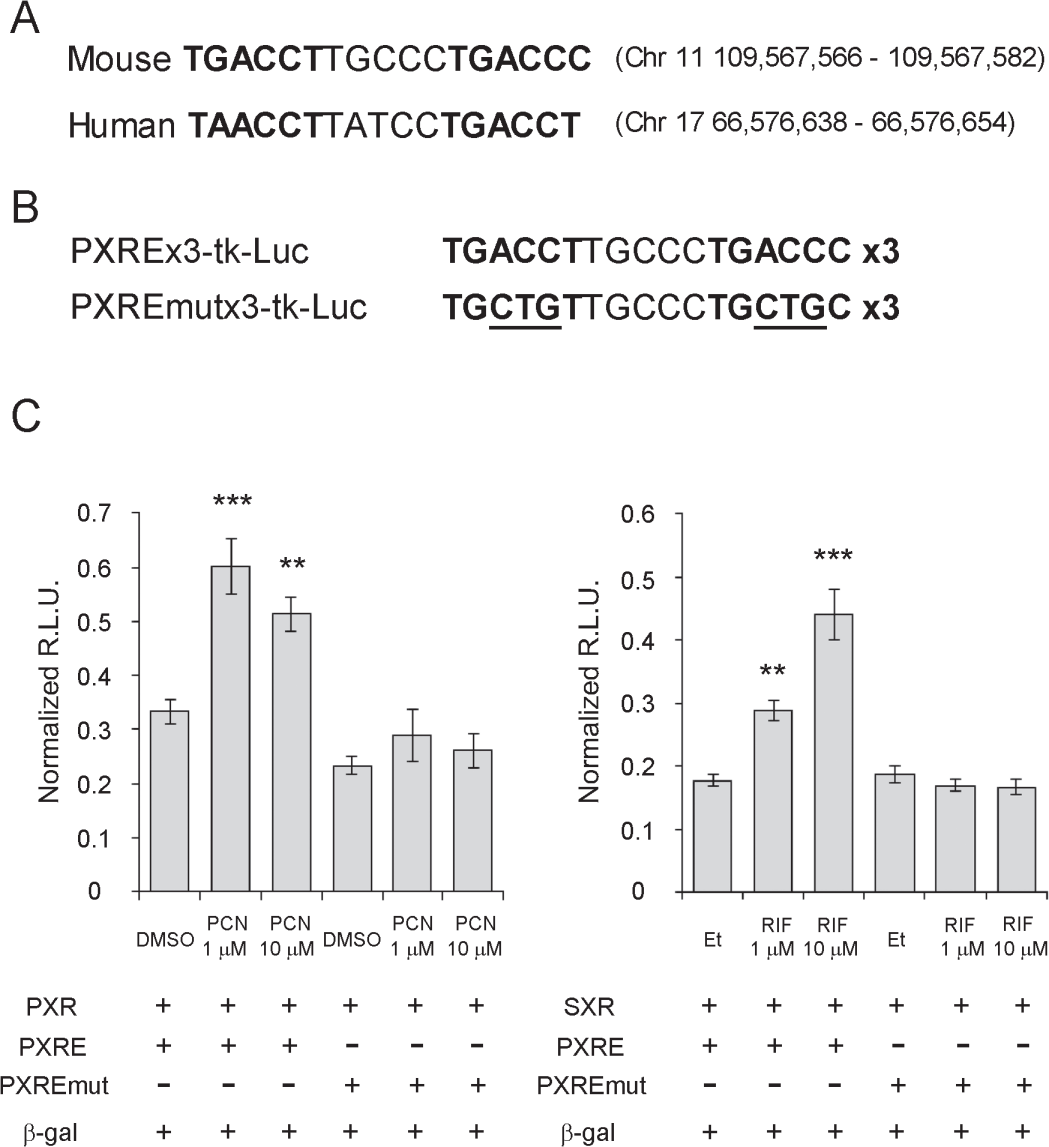
Consensus SXR/PXR responsive element motifs in the first introns of SXR and PXR genes. (A) Consensus SXR/PXR responsive element motifs, variant direct repeat 5, were identified in the first intron of both murine PXR gene (chromosome 11) and human SXR gene (chromosome 17). The bold letters indicate consensus SXR/PXR binding motif. (B) Generation of reporter plasmid containing three copies of PXR responsive element (PXRE) and PXRE with mutation (PXREmut). Underlined letters indicate mutated nucleotides. (C) Cos7 cells were transfected with PXR or SXR expression vector and reporter plasmid containing murine PXR responsive element or mutated PXR responsive element, and β-galactosidase expression vector (β-gal). The cells were then treated with indicated concentrations of PXR agonist pregnane-16α-carbonitrile (PCN) or SXR agonist rifampicin (RIF) or vehicles: DMSO for PCN and ethanol (Et) for RIF. Data are shown as relative light units (R.L.U.) normalized by β-galactosidase activity. ** P < 0.01, ***P < 0.001 in Dunnett’s test with vehicle treated group as a control.

We previously demonstrated that SXR is expressed in human primary chondrocytes at almost the same levels as in human primary osteoblasts [19]. Therefore, we hypothesized that SXR/PXR expressed in chondrocytes has a physiological role in maintaining articular cartilage and used the murine chondrocytic cell line ATDC5 for our initial investigations. Because ATDC5 cells express low amounts of endogenous PXR, we overexpressed human SXR in ATDC5 cells using an adenovirus vector. These cells were stimulated with the SXR ligands rifampicin, vitamin K2, or vehicle (ethanol) for 24 h. Gene expression was analyzed using microarrays, and hierarchical clustering analysis was performed. Clusters including SXR-dependent ligand-induced genes are shown in Fig. 3A and S1 Table. Among several candidate genes, induction of Fam20a (family with sequence similarity 20a) was validated by quantitative RT-PCR (Fig. 3B). A lower amount of Fam20a expression was detected in primary articular chondrocytes derived from PXR knockout mice compared with chondrocytes from wild-type mice (Fig. 3C), further supporting the SXR/PXR-dependent induction of Fam20a. We found a consensus SXR/PXR responsive element motif, variant direct repeat 5 [1], on the anti-sense strand in the first intron of both human and murine Fam20a genes (Fig. 4A). Transient transfection assays were performed using luciferase reporters containing three copies of the putative responsive element or three copies of a mutated element (Fig. 4B). Both the PXR agonist, pregnane-16α-carbonitrile (PCN), and the SXR agonist, rifampicin, increased the reporter gene activity in experiments using the wild-type element whereas they elicited no activity of the mutated element (Fig. 4C). This result indicated Fam20a is a primary responsive gene of SXR/PXR.

### Physiological role of Fam20a in chondrocytes

**Fig 5:**
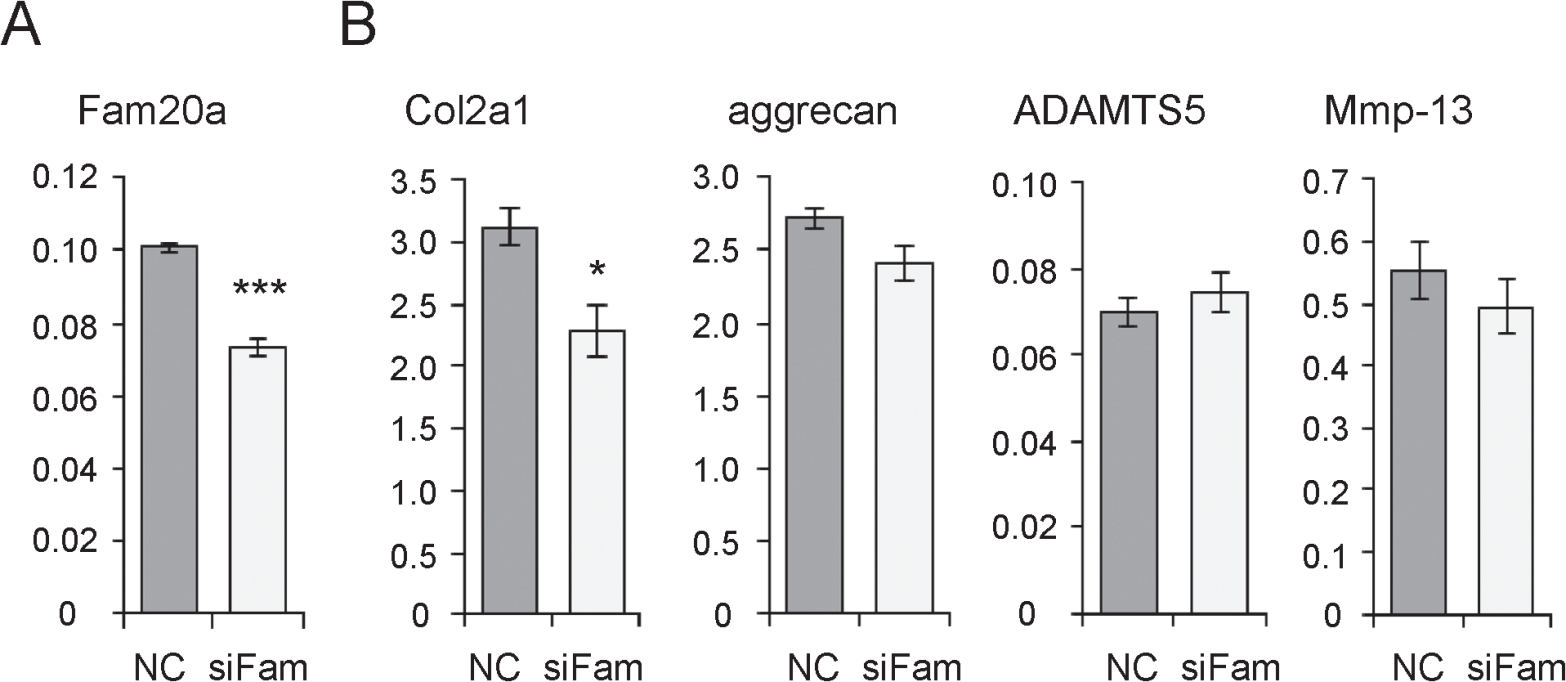
Physiological role of Fam20a in chondrocytes. (A) Expression of Fam20a was knocked down using siRNA (siFam) in primary murine articular chondrocytes. The effect of the siRNA was evaluated by comparison with primary murine chondrocytes transfected with scrambled-sequence siRNA as a negative control (NC). Expression of Fam20a was analyzed by quantitative real-time PCR. Expression of GAPDH was used as an internal control. ***P < 0.001. (B) Analysis of osteoarthritis-related genes in Fam20a knocked-down cells. Expression of Col2a1 and aggrecan was measured as major proteins comprising the extracellular matrix of articular chondrocytes. Expression of ADAMTS5 and Mmp-13 was measured as major enzymes that degrade the extracellular matrix. Expression of GAPDH was used as an internal control. A significant decrease of Col2a1 expression was observed in Fam20a knocked-down chondrocytes. *P < 0.05.

Fam20a is a secreted protein expressed in hematopoietic cells, lung, liver [20], and teeth [21]; however, its role in chondrocytes is unknown. To investigate the role of Fam20a in chondrocytes, expression of Fam20a in primary articular chondrocytes derived from WT C57BL6/J mice was knocked down using siRNA (Fig. 5A). Among the components comprising the extracellular matrix of articular cartilage, expression of Col2a1 was significantly decreased in Fam20a knocked-down cells, whereas expression of aggrecan was not changed (Fig. 5B). We also evaluated expression of proteinases responsible for articular cartilage degradation [22,23], ADAMTS5 (a disintegrin and metalloproteinase with thrombospondin motifs 5) and Mmp-13 (matrix metalloproteinase 13). Fam20 knockdown did not change expression of mRNAs encoding these proteases (Fig. 5B); therefore, we infer that Fam20a affects extracellular matrix biosynthesis in chondrocytes rather extracellular matrix degradation.

## Discussion

Here we showed that female PXR knockout mice display aging-dependent wearing of articular cartilage. These results indicate that SXR/PXR signaling has protective roles in cartilage tissue. We previously reported that SXR/PXR has protective roles in bone tissue [6,7]. It is interesting to note that SXR/PXR signaling thus affects both of the important components of skeletal tissue, bone and cartilage. Since our analysis is based on only female data, future studies would be needed to address whether the results are also applicable in male mice.

The width of articular cartilage could be influenced by formation of cartilage matrix, its degradation, and accumulation of physical damage. In our experiment, differences in cartilage width between PXR knockout mice and wild type mice were not evident until 8 months of age. This indicates a possible contribution of cumulative damage over time to this phenotype. It may also be possible that decreased production of cartilage matrix becomes more evident in aged PXRKO mice. We noticed that damage in the surface of the cartilage was milder than expected considering the decreased width of the cartilage in PXRKO mice and that the lesion is not identical to typical osteoarthritis. This may be because we are only observing age-related changes without interventions that cause joint instability such as ligament cutting.

SXR/PXR is expressed in human and murine primary chondrocytes; therefore, we hypothesized that SXR/PXR might have a physiological role in chondrocytes. We performed microarray analysis using ATDC5 cells overexpressing SXR and identified several candidate SXR responsive genes using hierarchical cluster analysis. The candidate genes were evaluated by quantitative real-time PCR; Fam20a was most strongly induced by both SXR agonists tested—rifampicin and vitamin K2. We found a consensus SXR/PXR responsive element motif in the first intron of both human and murine Fam20a genes and showed Fam20a is a primary responsive gene using reporter assays testing the identified SXR/PXR responsive element. Decreased expression of Fam20a in primary chondrocytes from PXRKO mice further supports our identification of Fam20a as an SXR responsive gene in chondrocyte. We note that these primary chondrocytes are derived from cartilage of newly born pups which do not reflect damaged cartilage in aged mice; thus, loss of Fam20a expression precedes the cartilage wearing phenotype. To understand the critical stage when expression of Fam20a is important for cartilage protection, further evaluation of Fam20a expression in both developing and aged pathological cartilage will be the subject of a future study.

Fam20a is a secreted protein that was originally described in hematopoietic cells [20]. Mutations in human FAM20A were shown to cause amelogenesis imperfecta and gingival hyperplasia [21]. Fam20a knockout mice were recently reported to have defects in the enamel layer of their teeth and ectopic calcification in arteries throughout their body [24]. Although the phenotypes of articular cartilage were not reported in humans or mice, Fam20a may be related to regulation of the extracellular matrix. Interestingly, Fam20b, which belongs to the same family as Fam20a, was shown to be expressed in chondrocytes in zebrafish and to play important roles in endochondral ossification [25]. Although no role for Fam20b in articular chondrocytes has yet been reported, these lines of evidence suggest that Fam20 family proteins also have some functions in the articular cartilage. We also noted decreased expression of Col2a1 in Fam20a knock-down chondrocytes. Since Fam20a is a secreted protein, it is conceivable that it regulates signaling from the extracellular matrix and affects cartilage turnover. We did not observe SXR-dependent induction of Fam20b or Fam20c in our microarray experiment, suggesting that SXR regulates only Fam20a among Fam20 family genes.

We previously showed that vitamin K2 functions as an agonist of SXR/PXR [5], independently of the previously known function of vitamin K as a co-factor for gamma carboxylation of substrate proteins [4]. The results of the current study suggest that activation of SXR/PXR is one of the mechanisms underlying the cartilage-protective effective of vitamin K shown by several epidemiological studies [10–12]. However, we cannot exclude the possibility that vitamin K activity mediated by gamma carboxylation of substrate proteins also plays role in articular cartilage. MGP (matrix gla protein) is a well-known substrate of gamma carboxylase in cartilage tissue [26]. Because MGP knockout mice die before the age of three months as a result of aortic rupture [26], the role of MGP in articular cartilage in aged mice remains unknown.

As in bone tissues, uncoupled turnover was shown to cause osteoarthritis based on serum levels of N-propeptide of type IIA procollagen (PIIANP) and urinary excretion of C-terminal crosslinking telopeptide of type II collagen (CTX-II) as cartilage specific turnover markers [27]. Considering the cartilage-protective effect shown in epidemiological studies and our results indicating that SXR/PXR signaling affects type II collagen synthesis, we can expect a therapeutic effect for at least some SXR agonists in osteoarthritis. However, to the best of our knowledge, the only randomized controlled study to date failed to show a therapeutic effect of vitamin K for treatment of patients with osteoarthritis [28]. That study was performed for three years, which might have been be too short an interval to affect cartilage turnover, or a more potent SXR/PXR agonist may be required to modify cartilage turnover in osteoarthritis. It is also possible that vitamin K has a disease-modifying effect, not in established osteoarthritis but rather in the developing phase when a preventive role can be still expected.

In conclusion, we show here for the first time that PXRKO mice displayed an osteoarthritis-like phenotype by performing histomorphometrical analyses of the articular cartilage. We identified Fam20a as a novel SXR/PXR responsive gene that is a candidate regulator of turnover in cartilage tissue. Although the clinical value of SXR agonists including vitamin K in osteoarthritis remains to be determined, our findings could help in the development of novel preventive and/or therapeutic strategies for osteoarthritis.

## Supporting Information

S1 Table Signal intensities of microarray data from the cluster including SXR-dependent ligand-induced genes. (DOCX)
