# Supplementary material for: Pregnane X Receptor Knockout Mice Display Aging-Dependent Wearing of Articular Cartilage
Source: PLoS One. 2015 Mar 6;10(3):e0119177. doi: 10.1371/journal.pone.0119177 (PMC4352085; doi:10.1371/journal.pone.0119177)
Supplement: S1 Table — (DOCX) [file pone.0119177.s001.docx]

| Gene | DsRed-Et | DsRed-VK | SXR-Et | SXR-RIF | SXR-VK |
| --- | --- | --- | --- | --- | --- |
| Ctgf | 9.752812 | 8.989222 | 9.904856 | 11.25310 | 9.618183 |
| Dpysl3 | 9.807649 | 8.844145 | 9.901917 | 11.02219 | 10.04685 |
| Ldhb | 9.301445 | 8.074899 | 9.508665 | 10.55324 | 9.591797 |
| Enpp3 | 7.012714 | 6.328574 | 7.594794 | 8.958472 | 7.936942 |
| Ldhb | 8.630983 | 7.906816 | 9.145033 | 10.18401 | 9.145033 |
| Mustn1 | 10.36434 | 9.363800 | 9.361790 | 12.00376 | 10.48344 |
| Grpr | 6.017932 | 5.876915 | 6.956408 | 8.569810 | 7.194998 |
| Itih2 | 6.570207 | 6.627729 | 6.831136 | 8.903056 | 7.921549 |
| Sfrp4 | 6.530573 | 6.793017 | 7.271904 | 8.752663 | 7.945208 |
| Crygs | 6.796296 | 7.701018 | 7.931015 | 9.089690 | 7.931015 |
| V1ra8 | 4.916196 | 5.846936 | 5.846936 | 6.324937 | 5.999162 |
| A930018M24Rik | 6.914937 | 6.409967 | 7.595124 | 8.558361 | 8.117041 |
| Fam20a | 8.566137 | 8.120222 | 9.562945 | 10.32813 | 10.38226 |
| Sh3rf2 | 7.952825 | 7.541726 | 9.325401 | 10.29311 | 10.15687 |
| Fabp5 | 8.740423 | 7.973246 | 9.749333 | 10.19334 | 10.57636 |
| Fabp5 | 8.769535 | 7.997516 | 9.795330 | 10.22492 | 10.61710 |
| Ngf | 8.151452 | 7.570871 | 8.837461 | 9.234212 | 9.650284 |
| Igfbp4 | 9.252062 | 8.772798 | 9.920918 | 10.64152 | 10.84059 |
| Pcdh19 | 7.572980 | 7.016217 | 8.638244 | 9.152576 | 8.885323 |
| Sema3a | 8.320839 | 7.257047 | 9.823558 | 10.29112 | 10.21621 |
| Slc4a4 | 8.539684 | 7.582607 | 9.154371 | 9.822707 | 9.543290 |
| Slco1a5 | 7.974223 | 6.576743 | 8.810938 | 9.617700 | 8.993071 |
| Gca | 7.100155 | 6.148714 | 6.958507 | 8.557863 | 8.190439 |
| Slc40a1 | 7.278267 | 6.431742 | 7.456552 | 8.580754 | 9.661776 |

Supplementary Table

Signal intensities of microarray data from the cluster including SXR-dependent ligand-induced genes.
